# Supplementary material for: Perinatal factors affecting platelet parameters in late preterm and term neonates
Source: PLoS One. 2020 Nov 19;15(11):e0242539. doi: 10.1371/journal.pone.0242539 (PMC7676724; doi:10.1371/journal.pone.0242539)
Supplement: S1 Table — (DOCX) [file pone.0242539.s001.docx]

**S1 Table. Factors affecting platelet parameters in late preterm and term neonates.**

|  | PCT (%) | |  | PDW (%) |  | MPV (fL) |  |  | PLT (×10^3^/μL) | |
| --- | --- | --- | --- | --- | --- | --- | --- | --- | --- | --- |
| (n) | univariate analysis  Median [IQR] | multivariate analysis  p-value (β) |  | univariate analysis  Median [IQR] |  | univariate analysis  Median [IQR] | multivariate analysis  p-value (β) |  | univariate analysis  Median [IQR] | multivariate analysis  p-value (β) |
| Male (207) | 0.25 [0.20-0.29] |  |  | 10.8 [10.0-12.3] |  | 9.7 [9.1-10.2] |  |  | 26.2 [21.2-30.5] |  |
| Female (193) | 0.26 [0.22-0.32] |  |  | 11.1 [10.3-12.4] |  | 9.9 [9.4-10.3] |  |  | 27.3 [22.6-32.7] |  |
|  | **P=0.012** | **P=0.001（-0.159）** |  | **P=0.049** |  | **P=0.043** | **P=0.011 (-0.124)** |  | **P=0.039** | **P= 0.011 (-0.124)** |
| RDS (31) | 0.26 [0.21-0.28] |  |  | 10.9 [10.1-13.1] |  | 9.9 [9.4-10.4] |  |  | 24.7 [22.8-31.9] |  |
| non-RDS (369) | 0.23 [0.22-0.26] |  |  | 10.9 [10.2-12.3] |  | 9.7 [9.3-10.2] |  |  | 26.7 [21.7-31.6] |  |
|  | P=0.123 |  |  | P=0.965 |  | P=0.139 |  |  | P=0.609 |  |
| SGA (109) | 0.24 [0.17-0.28] |  |  | 10.9 [10.3-12.7] |  | 9.9 [9.4-10.4] |  |  | 24.8 [17.1-30.2] |  |
| non-SGA (291) | 0.26 [0.22-0.30] |  |  | 10.9 [10.1-12.2] |  | 9.7 [9.3-10.2] |  |  | 27.0 [22.9-32.0] |  |
|  | **P=0.001** | **P=0.006（-0.139）** |  | P=0.248 |  | P=0.104 |  |  | **P=0.001** | **P=0.001 (-0.173)** |
| PROM (41) | 0.24 [0.21-0.31] |  |  | 10.9 [10.3-12.0] |  | 9.9 [9.4-10.3] |  |  | 25.0 [20.3-31.3] |  |
| non-PROM (359) | 0.25 [0.21-0.30] |  |  | 10.9 [10.2-12.4] |  | 9.7 [9.3-10.3] |  |  | 26.6 [21.9-31.8] |  |
|  | P=0.647 |  |  | P=0.851 |  | P=0.149 |  |  | P=0.390 |  |
| CAM (16) | 0.245 [0.21-0.29] |  |  | 11.2 [10.9-11.9] |  | 10.3 [10.0-10.6] |  |  | 25.0 [21.6-30.3] |  |
| non-CAM (384) | 0.26 [0.20-0.29] |  |  | 10.9 [10.2-12.4] |  | 9.7 [9.2-10.2] |  |  | 26.5 [21.9-31.7] |  |
|  | P=0.796 |  |  | P=0.282 |  | **P=0.001** | **P=0.001 (-0.173)** |  | P=0.553 |  |
| PA (14) | 0.22 [0.16-0.32] |  |  | 11.0 [10.0-13.5] |  | 9.85 [9.4-10.2] |  |  | 24.5 [17.6-32.6] |  |
| non-PA (386) | 0.25 [0.21-0.30] |  |  | 10.9 [10.2-12.4] |  | 9.8 [9.3-10.3] |  |  | 26.5 [21.9-31.6] |  |
|  | P=0.280 |  |  | P=0.770 |  | P=0.614 |  |  | P=0.444 |  |
| PIH (28) | 0.21 [0.16-0.27] |  |  | 11.0 [10.0-12.2] |  | 9.85 [9.4-10.2] |  |  | 21.7 [15.6-26.6] |  |
| non-PIH (372) | 0.26 [0.21-0.30] |  |  | 10.9 [10.2-12.4] |  | 9.8 [9.3-10.3] |  |  | 26.7 [22.1-31.9] |  |
|  | **P=0.003** | **P=0.017 (-0.120)** |  | P=0.963 |  | P=0.463 |  |  | **P=0.001** | **P=0.007 (-0.135)** |
| GA | r=-0.036 |  |  | r=-0.004 |  | r=0.014 |  |  | r=-0.064 |  |
|  | P=0.472 |  |  | P=0.937 |  | P=0.781 |  |  | P=0.200 |  |
| BW | r=0.080 |  |  | r=-0.024 |  | r=-0.020 |  |  | r=0.047 |  |
|  | P=0.112 |  |  | P=0.627 |  | P=0.693 |  |  | P=0.352 |  |
| AP 1 | r=0.028 |  |  | r=0.017 |  | r=-0.018 |  |  | r=0.027 |  |
|  | P=0.582 |  |  | P=0.738 |  | P=0.721 |  |  | P=0.585 |  |
| AP5 | r=0.044 |  |  | r=-0.001 |  | r=0.013 |  |  | r=0.051 |  |
|  | P=0.384 |  |  | P=0.983 |  | P=0.801 |  |  | P=0.305 |  |

**PCT, plateletcrit; PDW, platelet distribution width; MPV, mean platelet volume; PLT, platelet count; GA, gestational age; BW, birth weight; RDS, respiratory distress syndrome; SGA, small for gestational age; AP1, Apgar score at 1 min; AP5, Apgar score at 5 min; PROM, premature rupture of membranes; CAM, chorioamnionitis; PA, placental abruption; PIH, pregnancy-induced hypertension; IQR, median interquartile range. Significant correlation between GA, BW, Apgar score and platelet parameters were analyzed using Spearman’s rank correlation (r). β means standardized regression coefficient.**
