# Supplementary material for: Perinatal factors affecting platelet parameters in late preterm and term neonates
Source: PLoS One. 2020 Nov 19;15(11):e0242539. doi: 10.1371/journal.pone.0242539 (PMC7676724; doi:10.1371/journal.pone.0242539)
Supplement: S2 Table — (DOCX) [file pone.0242539.s002.docx]

**S2 Table. Platelet parameters in SGA and not neonates.**

|  |  | Late Preterm (n=142) | |  |  | Term (n=258) | | |  | Total (n=400) | | |
| --- | --- | --- | --- | --- | --- | --- | --- | --- | --- | --- | --- | --- |
|  |  | SGA (n=57) | non-SGA (n=85) | p-value |  | SGA (n=52) | non-SGA (n=206) | p-value |  | SGA (n=109) | non-SGA (n=291) | p-value |
| GA (weeks) [IQR] |  | 36.1 [35.4-36.2] | 35.8 [35.4-36.2] | **0.026** |  | 38.0 [37.4-38.7] | 38.2 [37.5-39.5] | **0.049** |  | 36.8 [36.1-38.0] | 37.8 [36.4-38.7] | **<0.001** |
| BW (gram) [IQR] |  | 1754 [1545-1903] | 2504 [2244-2782] | **<0.001** |  | 2090 [1850-2370] | 2911 [2638-3205] | **<0.001** |  | 1858 [1655-2110] | 2776 [2534-3104] | **<0.001** |
| PCT (%) [IQR] |  | 0.24 [0.17-0.28] | 0.26 [0.22-0.31] | **0.020** |  | 0.23 [0.16-0.29] | 0.26 [0.22-0.30] | **0.024** |  | 0.24 [0.17-0.28] | 0.26 [0.22-0.30] | **0.001** |
| PDW [IQR] |  | 10.9 [10.1-13.5] | 11.0 [10.0-12.5] | 0.533 |  | 11.0 [10.4-12.5] | 10.9 [10.9-12.1] | 0.299 |  | 10.9 [10.3-12.7] | 10.9 [10.1-12.2] | 0.248 |
| MPV (fl) [IQR] |  | 9.9 [9.2-10.4] | 9.8 [9.1-10.2] | 0.454 |  | 10.0 [9.4-10.4] | 9.7 [9.3-10.3] | 0.193 |  | 9.9 [9.4-10.4] | 9.7 [9.3-10.2] | 0.104 |
| PLT (×103/μL) [IQR] |  | 24.4 [16.9-29.8] | 28.0 [23.8-32.8] | **0.010** |  | 24.8 [17.4-30.5] | 26.7 [22.3-31.8] | **0.023** |  | 24.8 [17.1-30.2] | 27.0 [22.9-32.0] | **0.001** |
| Male (%) |  | 25 (49%) | 51 (60%) | 0.059 |  | 23 (44%) | 108 (52.4) | 0.184 |  | 48 (44%) | 159 (52.4) | 0.059 |
| PIH (%) |  | 16 (28%) | 8 (9.4%) | **0.004** |  | 2 (3.8%) | 2 (1.0%) | 0.182 |  | 18 (3.8%) | 10 (1.0%) | **<0.001** |

**MPV, mean platelet volume; PDW, platelet distribution width; MPV, mean platelet volume; PLT, platelet; PCT, plateletcrit; PIH, pregnancy-induced hypertension; SGA, small for gestational age; IQR, median interquartile range.**
