# Supplementary material for: Perinatal factors affecting platelet parameters in late preterm and term neonates
Source: PLoS One. 2020 Nov 19;15(11):e0242539. doi: 10.1371/journal.pone.0242539 (PMC7676724; doi:10.1371/journal.pone.0242539)
Supplement: Attachment — Submitted filename: Summary of Comments on PONE-D-19-33766_reviewer_Bhatti comments.pdf [file pone.0242539.s004.pdf]

PLOS ONE

Perinatal factors affecting platelet parameters in late preterm and term neonates.

--Manuscript Draft--

**Manuscript Number:**	PONE-D-19-33766
**Article Type:**	Research Article
**Full Title:**	Perinatal factors affecting platelet parameters in late preterm and term neonates.
**Short Title:**	Platelet parameters in late and term neonates
**Corresponding Author:**	Hayato Go<br>Fukushima Kenritsu Ika Daigaku Fuzoku Byoin<br>Fukushima, JAPAN
**Keywords:**	platelet parameters, mean platelet volume, late preterm neonates
**Abstract:**	Objectives<br>To investigate perinatal factors affecting platelet parameters in late preterm and term neonates.<br>Study Design<br>We retrospectively investigated platelet parameters including platelet counts (PLT), plateletcrit (PCT), mean platelet volume (MPV), and platelet distribution width (PDW) on the first day of life in 134 late preterm and 357 term neonates admitted to our NICU from 2006 through 2017.<br>Results<br>PLT, MPV, PCT, PDW on the first day did not significantly differ between the two groups.<br>In term neonates, multivariate analysis revealed that PCT correlated with small for gestational age (SGA) (β =-0.135, P =0.002) and Apgar score at 5 min (β = 0.114, P = 0.032).PLT was associated with SGA (β =-0.225, P =0.001), Apgar score at 5 min (β = 0.025, P = 0.008) and pregnancy-induced hypertension PIH (β = -0.112, P =0.033).<br>In late preterm neonates, multivariate analysis revealed that PLT and PCT were associated with SGA, whereas no factors associated with PDW and MPV were found.<br>In all patients studied, chorioamniotitis (CAM) was significantly associated with MPV (CAM=10.4 fl vs. no CAM=9.6 fl, P <0.001). Multivariate analysis showed that SGA was associated with PCT and PDW. Furthermore, SGA (β =-0.252, P <0.001) and Apgar score at 5 min. (β = 0.096, P =0.031) were associated with PLT.<br><br>Conclusion<br>This study demonstrates that different maternal and neonatal complications affect platelet parameters in late preterm and term neonates.
**Order of Authors:**	Hayato Go
	Hitoshi Ohto
	Kenneth E Nollet
	Nozomi Kashiwabara
	Mina Chisiki
	Masato Hoshino
	Kei Ogasawara
	Yukihiko Kawasaki
	Nobuo Momoi
	Mitsuaki Hosoya
**Additional Information:**	
**Question**	**Response**
**Financial Disclosure**	NO - Include this sentence at the end of your statement: The funders had no role in

Powered by Editorial Manager® and ProduXion Manager® from Aries Systems Corporation

Summary of Comments on PONE-D-19-33766_reviewer_Bhatti comments.pdf

This page contains no comments

Enter a financial disclosure statement that describes the sources of funding for the work included in this submission. Review the submission guidelines for detailed requirements. View published research articles from *PLOS ONE* for specific examples.<br><br>This statement is required for submission and **will appear in the published article** if the submission is accepted. Please make sure it is accurate.<br><br>**Unfunded studies**<br>Enter: *The author(s) received no specific funding for this work.*<br><br>**Funded studies**<br>Enter a statement with the following details:<br>• Initials of the authors who received each award<br>• Grant numbers awarded to each author<br>• The full name of each funder<br>• URL of each funder website<br>• Did the sponsors or funders play any role in the study design, data collection and analysis, decision to publish, or preparation of the manuscript?<br>• **NO** - Include this sentence at the end of your statement: *The funders had no role in study design, data collection and analysis, decision to publish, or preparation of the manuscript.*<br>• **YES** - Specify the role(s) played.<br><br>* typeset	study design, data collection and analysis, decision to publish, or preparation of the manuscript.
**Competing Interests**<br><br>Use the instructions below to enter a competing interest statement for this submission. On behalf of all authors, disclose any competing interests that could be perceived to bias this work—acknowledging all financial support and any other relevant financial or non-financial competing interests.<br><br>This statement **will appear in the published article** if the submission is accepted. Please make sure it is accurate. View published research articles	The authors have declared that no competing interests exist.

This page contains no comments

from *PLOS ONE* for specific examples.<br><br>**NO authors have competing interests**<br><br>Enter: *The authors have declared that no competing interests exist.*<br><br>**Authors with competing interests**<br><br>Enter competing interest details beginning with this statement:<br><br>*I have read the journal's policy and the authors of this manuscript have the following competing interests: [insert competing interests here]*<br><br>* typeset	
**Ethics Statement**<br><br>Enter an ethics statement for this submission. This statement is required if the study involved:<br><br>• Human participants<br>• Human specimens or tissue<br>• Vertebrate animals or cephalopods<br>• Vertebrate embryos or tissues<br>• Field research<br><br>Write "N/A" if the submission does not require an ethics statement.<br><br>General guidance is provided below. Consult the submission guidelines for detailed instructions. **Make sure that all information entered here is included in the Methods section of the manuscript.**	The Ethics Committee of FMU, guided by local policy, national law, and the World Medical Association Declaration of Helsinki, approved this study without requiring informed consent from guardians.

This page contains no comments

**Format for specific study types**<br><br>**Human Subject Research (involving human participants and/or tissue)**<br>• Give the name of the institutional review board or ethics committee that approved the study<br>• Include the approval number and/or a statement indicating approval of this research<br>• Indicate the form of consent obtained (written/oral) or the reason that consent was not obtained (e.g. the data were analyzed anonymously)<br><br>**Animal Research (involving vertebrate animals, embryos or tissues)**<br>• Provide the name of the Institutional Animal Care and Use Committee (IACUC) or other relevant ethics board that reviewed the study protocol, and indicate whether they approved this research or granted a formal waiver of ethical approval<br>• Include an approval number if one was obtained<br>• If the study involved *non-human primates*, add *additional details* about animal welfare and steps taken to ameliorate suffering<br>• If anesthesia, euthanasia, or any kind of animal sacrifice is part of the study, include briefly which substances and/or methods were applied<br><br>**Field Research**<br><br>Include the following details if this study involves the collection of plant, animal, or other materials from a natural setting:<br>• Field permit number<br>• Name of the institution or relevant body that granted permission	
**Data Availability**<br><br>Authors are required to make all data underlying the findings described fully available, without restriction, and from the time of publication. PLOS allows rare exceptions to address legal and ethical concerns. See the PLOS Data Policy and FAQ for detailed information.	No - some restrictions will apply

This page contains no comments

A Data Availability Statement describing where the data can be found is required at submission. Your answers to this question constitute the Data Availability Statement and **will be published in the article**, if accepted.<br><br>**Important:** Stating 'data available on request from the author' is not sufficient. If your data are only available upon request, select 'No' for the first question and explain your exceptional situation in the text box.<br><br>Do the authors confirm that all data underlying the findings described in their manuscript are fully available without restriction?	
**Describe where the data may be found in full sentences. If you are copying our sample text, replace any instances of XXX with the appropriate details.**<br><br>• If the data are **held or will be held in a public repository**, include URLs, accession numbers or DOIs. If this information will only be available after acceptance, indicate this by ticking the box below. For example: *All XXX files are available from the XXX database (accession number(s) XXX, XXX.).*<br>• If the data are all contained **within the manuscript and/or Supporting Information files**, enter the following: *All relevant data are within the manuscript and its Supporting Information files.*<br>• If neither of these applies but you are able to provide **details of access elsewhere**, with or without limitations, please do so. For example:<br><br>*Data cannot be shared publicly because of [XXX]. Data are available from the XXX Institutional Data Access / Ethics Committee (contact via XXX) for researchers who meet the criteria for access to confidential data.*<br><br>*The data underlying the results presented in the study are available from (include the name of the third party*	This text is appropriate if the data are owned by a third party and authors do not have permission to share the data.

This page contains no comments

and contact information or URL).<br>• This text is appropriate if the data are owned by a third party and authors do not have permission to share the data.<br><br>* typeset	
Additional data availability information:	Tick here if your circumstances are not covered by the questions above and you need the journal's help to make your data available.

Powered by Editorial Manager® and ProduXion Manager® from Aries Systems Corporation

This page contains no comments

Manuscript

Click here to download Manuscript platelet indices in lete preterm 1206 PLoS One.docx

Title

Perinatal factors affecting platelet parameters in late preterm and term neonates.

Hayato Go[1], Hitoshi Ohto[2], Kenneth E. Nollet[3], Nozomi Kashiwabara[1], Mina Chishiki[1], Masato Hoshino[1], Kei Ogasawara[1], Yukihiko Kawasaki[4], Nobuo Momoi[1], Mitsuaki Hosoya[1]

[1]Department of Pediatrics, Fukushima Medical University School of Medicine, Fukushima, Japan

[2]Department of Advanced Cancer Immunotherapy, Fukushima Medical University School of Medicine, Fukushima, Japan

[3]Department of Blood Transfusion and Transplantation Immunology, Fukushima Medical University School of Medicine, Fukushima, Japan

[4]Department of Pediatrics, Sapporo Medical University School of Medicine, Hokkaido, Japan

This page contains no comments

Corresponding Author: Hayato Go, MD, PhD

Department of Pediatrics, Fukushima Medical University School of Medicine, Fukushima, 960-1295, Japan

Tel: +81-245471111; Fax: +81-245482177

E-mail: gohayato2525@gmail.com

Conflict of interest: Nothing to declare

Number of Tables and Figures: 4 Tables and 1 Figure

This page contains no comments

Running title: Platelet parameters in late and term neonates

Keywords： platelet parameters, mean platelet volume, late preterm neonates

This page contains no comments

Abstract

Objectives

To investigate perinatal factors affecting platelet parameters in late preterm and term neonates.

Study Design

We retrospectively investigated platelet parameters including platelet counts (PLT), plateletcrit (PCT), mean platelet volume (MPV), and platelet distribution width (PDW) on the first day of life in 134 late preterm and 357 term neonates admitted to our NICU from 2006 through 2017.

Results

PLT, MPV, PCT, PDW on the first day did not significantly differ between the two groups.

In term neonates, multivariate analysis revealed that PCT correlated with small for gestational age (SGA) (β=-0.135, *P*=0.002) and Apgar score at 5 min (β=0.114, *P* = 0.032).PLT was associated with SGA (β=-0.225, *P*=0.001), Apgar score at 5 min (β=0.025, *P* = 0.008) and pregnancy-induced hypertension PIH (β=-0.112, *P*=0.033).

This page contains no comments

In late preterm neonates, multivariate analysis revealed that PLT and PCT were associated with SGA, whereas no factors associated with PDW and MPV were found. In all patients studied, chorioamniotitis (CAM) was significantly associated with MPV (CAM=10.4 fl vs. no CAM=9.6 fl, $P<0.001$). Multivariate analysis showed that SGA was associated with PCT and PDW. Furthermore, SGA ($\beta=-0.252$, $P<0.001$) and Apgar score at 5 min. ($\beta=0.096$, $P=0.031$) were associated with PLT.

Conclusion

This study demonstrates that different maternal and neonatal complications affect platelet parameters in late preterm and term neonates.

This page contains no comments

Introduction

Platelets parameters are associated with various physiological and pathological functions in various disease diseases (1,2,3). Platelet production is a complex process arising from the proliferation and differentiation of megakaryocytes under the stimulating influence of thrombopoietin. Platelet function in premature neonates is immature. Previous research indicated that low birth weight infants were at almost 2.5-fold increased risk for thrombocytopenia (4). On the other hand, elevated mean platelet volume (MPV), platelet distribution width (PDW), and increased plateletcrit (PCT) are associated with neonatal disease such as sepsis, fungal infection and intraventricular hemorrhage (5, 6, 7, 8). Among extremely low birth weight neonates born to mothers with preeclampsia, the MPV/platelet count (PLT) ratio at birth significantly correlated with mortality (9). Also, changes of PDW during the neonatal period were associated with thrombocytopenia with neonatal sepsis in very low birth weight infants (10). However, few studies have addressed whether perinatal factors may be associated with platelet parameters (PCT, PLT, MPV and PDW) at birth in a large cohort of late preterm and term neonates. The objective of this study was to investigate the factors affecting platelet parameters at birth in late preterm and term neonates.

Number: 1 Author: faizahbhatti Subject: Sticky Note Date: 1/28/20, 11:19:49 AM

The opening sentence of the Introduction is very vague and not compelling. Please be more specific and deliberate in your writing.

Number: 2 Author: faizahbhatti Subject: Sticky Note Date: 1/28/20, 10:38:53 AM

What is your hypothesis? Its not clear what you are adding to the existing body of knowledge.

Number: 3 Author: faizahbhatti Subject: Sticky Note Date: 1/28/20, 10:40:05 AM

What factors? What comparisons? What are the variables you are interested in studying. At this point, this sounds like an observational study, so please state that explicitly.

Number: 4 Author: faizahbhatti Subject: Sticky Note Date: 1/28/20, 10:44:23 AM

Why was analysis on preterm babies not performed? That data is discussed in the introduction, but you do not provide a rationale for why it is not included in this study. Why did yo limit to term and late preterm?

Methods

Study design and population

This retrospective, single center, cohort study examined records from 2006 through 2017 at the neonatal intensive care unit (NICU) of Fukushima Medical University Hospital (FMU). The Ethics Committee of FMU, guided by local policy, national law, and the World Medical Association Declaration of Helsinki, approved this study without requiring informed consent from guardians. Of 1517 neonates admitted to our NICU between January 2006 and December 2017, there were 491 born at term or late preterm (weeks 35-36). Late preterm neonates and full term neonates numbered 134 and 357, respectively. Exclusion criteria were congenital anomalies, neonatal alloimmune thrombocytopenia, and neonates not tested within 12 hours of birth.

Platelet parameter measurements [1]

Blood samples were collected through umbilical cord or peripheral venipuncture to measure PLT, PCT, MPV and PDW of each newborn. Complete blood counts were measured using a Sysmex CS-5100 coagulation analyzer (Sysmex, Kobe, Japan) on admission. [2]

Number: 1 Author: faizahbhatti Subject: Sticky Note Date: 1/28/20, 10:52:41 AM

When were these samples collected? Beginning of Day of Life 1? At the end? Any time in the first 24 hours? This will confound your results significantly.

Number: 2 Author: faizahbhatti Subject: Sticky Note Date: 1/28/20, 11:06:58 AM

One of the factors you have failed to examine is how many babies required resucitation at birth with epinephrine? Epinephrine has been reported to cause platelet aggregation as well as recruitment into the circulation. See Kjeldsen SE, Weder AB, Egan B, Neubig R, Zweifler AJ, Julius S. Effect of circulating epinephrine on platelet function and hematocrit. *Hypertension*. 1995;25(5):1096–1105.

Furthermore, reactivity to epinephrine via ADP and thromboxane differs between neonates and adults. Rajasekhar D, Barnard MR, Bednarek FJ, Michelson AD. Platelet hyporeactivity in very low birth weight neonates. *Thromb Haemost*. 1997;77(5):1002–1007.

Therefore it is important to note what the exposure to resucitation and epinephrine is in these subjects.

Prenatal and postnatal risk factors

Platelet parameters were compared with demographic variables including gender, birth weight (BW), and gestational age (GA). Furthermore, we considered pregnancy-induced hypertension (PIH), chorioamnionitis (CAM), premature rupture of membranes (PROM), and placental abruption (PA) as possible prenatal risk factors, and small for gestational age (SGA, birth weight <10th percentile), appropriate for gestational age (AGA, birth weight >10th percentile), respiratory distress syndrome (RDS), and Apgar score as factors possibly associated with platelet parameters.

Statistical analysis

Platelet parameters from medical records were rendered as medians of non-normally distributed continuous variables. The Mann–Whitney *U*-test was performed to analyze nonparametric data. Likewise, correlations between BW, GA, and Apgar score versus

Page: 14

Number: 1 Author: faizahbhatti Subject: Sticky Note Date: 1/28/20, 10:41:44 AM

It would have been nice if platelet morphology was examined via microscopy on peripheral smears

platelet parameters were also investigated using Spearman’s rank correlation (*r*). Moreover, the variables at $P < 0.05$ in univariate analysis were entered into a multiple regression analysis to identify independent prognostic factors.

SPSS for Mac, release 25.0 (SPSS, Chicago, IL) was used for statistical analyses with $P < 0.05$ considered to be statistically significant.

Results

Table 1 shows platelet parameters in late preterm and term neonates. There are no significant differences in platelet parameters between late preterm and term neonates. Figures 1A and 1B show significant correlation between BW and PLT, but none between GA and PLT. Likewise, Figures 1C and 1D show significant correlation between BW and PCT, but none between GA and PCT.

Table 1. Platelet parameters in late preterm and term neonates

	Late preterm (n=134)	Term (n=357)	p-value
GA (weeks)	36.0	38.2	<0.001
BW (grams)	2234	2758	<0.001
PCT (%)	0.24	0.25	0.492
PDW	11.3	11.3	0.701
MPV (fl)	9.8	9.8	0.879

Page: 15

Number: 1 Author: faizahbhatti Subject: Sticky Note Date: 1/28/20, 10:47:16 AM

Here it would be very important to separate the term and late preterm subjects by AGA and SGA status. So have four columns instead of two.

PLT ($\times 10^3/\mu L$)	25.6	25.6	0.973

GA, gestational age; BW, birth weight; PCT, plateletcrit; PDW, platelet distribution width; MPV, mean platelet volume; PLT, platelet count.

Tables 2 and 3 outline the results of univariate and multivariate analyses for the correlation between perinatal factors and platelet parameters in preterm and term neonates, respectively.

As shown in Table 2, in term neonates, MPV and PDW were associated with CAM (CAM=10.4 fl vs. no CAM=9.6 fl, *P* = 0.001) and PROM (PROM=10.3 % vs. no PROM=11.3 %, *P*=0.049), respectively. Multivariate analysis revealed that PCT correlated with SGA (β=-0.135, *P*=0.002) and Apgar score at 5 min (β=0.114, *P* = 0.032). PLT was associated with SGA (β=-0.225, *P*=0.001), Apgar score at 5 min (β=0.025, *P* = 0.008) and PIH (β=-0.112, *P*=0.033).

As shown in Table 3, univariate analysis revealed that PDW was correlated with PIH in late preterm neonates (PIH=10.4 fl vs. no PIH=11.3 fl, *P* = 0.038). Multivariate analysis revealed that PLT and PCT were associated with SGA, whereas no factors associated with PDW and MPV were found in late preterm neonates.

Table 2. Factors affecting platelet parameters in term neonates.

This page contains no comments

		PCT (%)		PDW (%)	MPV (fl)	PLT (×10^3/μL)	
		univariate analysis	multivariate analysis	univariate analysis	univariate analysis	univariate analysis	multivariate analysis
Male (n=182)	Yes	0.24		11.2	9.7	25.0	
	No	0.25		11.4	9.9	26.0	
	p-value	0.218		0.49	0.694	0.113	
RDS (n=26)	Yes	0.23		11.1	9.8	22.3	
	No	0.25		11.3	9.8	25.6	
	p-value	0.305		0.789	0.545	0.487	
SGA (n=50)	Yes	0.21		11.9	10	20.7	
	No	0.25		11.2	9.7	25.9	
	p-value (β)	0.000	**0.002 (-0.135)**	0.055	0.161	0.000	**0.001 (-0.225)**
Death (n=19)	Yes	0.24		11.8	10.3	23.2	
	No	0.25		11.2	9.7	25.5	
	p-value	0.381		0.062	0.063	0.099	
PROM (n=10)	Yes	0.22		10.3	9.6	23.9	
	No	0.25		11.3	9.8	25.6	
	p-value	0.226		0.049	0.436	0.254	
CAM (n=9)	Yes	0.21		11.4	10.4	21.4	
	No	0.25		11.4	9.6	26.1	
	p-value	0.584		0.125	0.001	0.121	
PA (n=5)	Yes	0.21		11.1	10.2	23.1	
	No	0.25		11.3	9.7	25.5	
	p-value	0.13		0.897	0.829	0.469	

Number: 1 Author: faizahbhatti Subject: Sticky Note Date: 1/28/20, 10:46:26 AM

What is your definition of SGA?

PIH (n=7)	Yes	0.19		10.9	9.7	20.1	
	No	0.50		11.3	9.8	25.6	
	p-value (β)	0.003	0.050 (-0.104)	0.242	0.618	0.025	**0.033 (-0.112)**
GA (week)	r	-0.039		-0.044	0.077	-0.063	
	p-value	0.469		0.419	0.149	0.236	
BW (gram)	r	0.130		-0.099	0.003	0.108	
	p-value (β)	0.014	0.726 (0.023)	0.063	0.952	0.041	0.821 (-0.015)
AP1	r	0.096		-0.087	-0.054	0.113	
	p-value	0.074		0.347	0.320	0.036	0.167 (-0.139)
AP5	r	0.132		-0.150	-0.079	0.153	
	p-value (β)	0.014	**0.032 (0.114)**	0.148	0.144	0.006	**0.008 (0.265)**

PCT, platelet count; PDW, platelet distribution width; MPV, mean platelet volume; PLT, platelet count; GA, gestational age; BW, birth weight; RDS, respiratory distress syndrome; SGA, small for gestational age; AP1, Apgar score at 1 min.; AP5, Apgar score at 5 min.; PROM, premature rupture of membranes; CAM, chorioamnionitis; PA, placental abruption; PIH, pregnancy-induced hypertension. Significant correlation between GA, BW, Apgar score and platelet parameters were analyzed using Spearman's rank correlation (r). β means standardized r[illegible]ression coefficient.

Table 3. Factors affecting platelet parameters in late preterm neonates.

Number: 1 Author: faizahbhatti Subject: Sticky Note Date: 1/28/20, 10:49:31 AM

You have omitted definitions for several of these variables. Is CAM clinical or culture proven? What is the definition of clinical chorio at your institution?

		PCT (%)		PDW (%)	MPV (fl)	PLT (×10³/μL)	
		univariate analysis	multivariate analysis	univariate analysis	univariate analysis	univariate analysis	multivariate analysis
Male (n=75)	Yes	0.24		11.2	9.8	24.9	
	No	0.24		11.2	9.9	25.8	
	p-value	0.569		0.971	0.217	0.905	
RDS (n=14)	Yes	0.22		11.2	9.7	23.6	
	No	0.24		11.0	9.9	25.8	
	p-value	0.101		0.434	0.366	0.243	
SGA (n=50)	Yes	0.20		11.7	10	22.1	
	No	0.25		11.2	9.8	27.1	
	p-value (β)	**0.002**	**0.024 (-0.265)**	0.119	0.296	**0.001**	**0.019 (-0.275)**
Death (n=6)	Yes	0.24		14.9	9.5	23.6	
	No	0.24		11.2	9.7	25.6	
	p-value	0.640		0.173	0.897	0.407	
PROM (n=16)	Yes	0.25		11.5	10.1	25.2	
	No	0.24		11.2	9.8	25.6	
	p-value (β)	0.226		0.500	**0.028 (0.065)**	0.511	
CAM (n=9)	Yes	0.26		11.2	10.3	28.0	
	No	0.24		11.5	9.8	26.0	
	p-value	0.448		0.427	0.233	0.297	
PA (n=10)	Yes	0.24		11.0	9.9	24.9	
	No	0.24		11.2	9.8	25.5	
	p-value	0.780		0.579	0.258	0.936	

This page contains no comments

PIH (n=8)	Yes	0.23		10.4	9.2	24.1	
	No	0.24		11.3	9.9	25.6	
	p-value (β)	0.573		**0.038**	0.049 (0.378)	0.511	
GA (week)	r	-0.007		-0.037	0.074	-0.003	
	p-value	0.938		0.974	0.343	0.974	
BW (gram)	r	0.202		-0.099	-0.071	0.206	
	p-value (β)	0.020	0.990 (0.002)	0.667	0.417	0.017	0.867
AP1	r	-0.005		0.063	0.025	0.028	
	p-value	0.954		0.468	0.775	0.751	
AP5	r	-0.025		0.042	0.071	-0.005	
	p-value	0.777		0.627	0.416	0.952	

PCT, platelet count; PDW, platelet distribution width; MPV, mean platelet volume; PLT, platelet count; GA, gestational age; BW, birth weight; RDS, respiratory distress syndrome; SGA, small for gestational age; AP1, Apgar score at 1 min.; AP5, Apgar score at 5 min.; PROM, premature rupture of membranes; CAM, chorioamnionitis; PA, placental abruption; PIH, pregnancy-induced hypertension. Significant correlation between GA, BW, Apgar score and platelet parameters were analyzed using Spearman's rank correlation (r). β means standardized regression coefficient.

When all patients were studied, CAM was significantly associated with MPV ((CAM=10.4 fl vs. no CAM=9.6 fl, P<0.001).

Multivariate analysis showed that SGA was associated with PCT and PDW. Furthermore, SGA (β=-0.252, P<0.001) and Apgar score at 5 min. (β=0.096, P=0.031) were associated with PLT (Table 4).

This page contains no comments

Next, we compared the differences of platelet parameters between SGA and AGA (Table 5). Table 5 showed that PLT, and PCT were significantly decreased and PDW was significantly increased in SGA neonates compared with AGA neonates.

Table 4. Factors affecting platelet parameters in late preterm and term neonates.

		PCT (%)		PDW (%)		MPV (fl)	PLT ($\times 10^3$/μL)	
		univariate analysis	multivariate analysis	univariate analysis	univariate analysis	univariate analysis	univariate analysis	multivariate analysis
Male (n=259)	Yes	0.24		11.2		9.7	25.4	
	No	0.25		11.4		9.9	26.6	
	p-value	0.193		0.561		0.293	0.213	
RDS (n=40)	Yes	0.25		11.1		9.7	23.3	
	No	0.22		11.3		9.8	25.6	
	p-value	0.073		0.525		0.975	0.229	
SGA (n=87)	Yes	0.21		11.6		9.9	22.1	
	No	0.25		11.2		9.8	25.9	
	p-value(β)	**<0.001**	**0.004（-0.265）**	**0.016**	**0.003 (0.137)**	0.077	**<0.001**	**<0.001 (-0.252)**
Death (n=25)	Yes	0.24		11.8		10.3	26.5	
	No	0.24		11.2		9.8	25.6	
	p-value	0.381		0.062		0.114	0.099	
PROM (n=26)	Yes	0.24		10.9		9.8	23.9	
	No	0.24		11.3		9.9	25.6	
	p-value	0.826		0.513		0.225	0.826	
CAM (n=19)	Yes	0.21		11.4		10.4	21.4	

This page contains no comments

	No	0.25		11.4		9.6	26.1	
	p-value	0.842		0.056		**<0.001**	0.163	
PA (n=15)	Yes	0.21		11.1		10.2	23.1	
	No	0.25		11.3		9.7	25.5	
	p-value	0.497		0.558		0.372	0.615	
PIH (n=15)	Yes	0.20		10.9		9.7	20.1	
	No	0.24		11.3		9.8	25.6	
	p-value(β)	**0.012**	0.089(-0.076)	**0.019**	0.138 (-0.068)	0.071	0.058	
GA (week)	r	-0.039		-0.044		0.077	-0.063	
	p-value	0.891		0.862		0.562	0.319	
BW (gram)	r	0.130		-0.099		0.003	0.108	
	p-value(β)	**0.002**	0.766(0.017)	0.280		0.655	**0.015**	0.781 (-0.015)
AP1	r	0.096		-0.087		-0.054	0.113	
	p-value	0.135		0.334		0.475	0.052	
AP5	r	0.132		-0.150		-0.079	0.153	
	p-value(β)	0.069		**0.041**	0.316 (-0.046)	0.421	**0.020**	**0.031 (0.096)**

PCT, plateletcrit; PDW, platelet distribution width; MPV, mean platelet volume; PLT, platelet count; GA, gestational age; BW, birth weight; RDS, respiratory distress syndrome; SGA, small for gestational age; AP1, Apgar score at 1 min; AP5, Apgar score at 5 min; PROM, premature rupture of membranes; CAM, chorioamnionitis; PA, placental abruption; PIH, pregnancy-induced hypertension. Significant correlation between GA, BW, Apgar score and platelet parameters were analyzed using Spearman's rank correlation (r). β means standardized regression coefficient.

This page contains no comments

Table 5. Platelet parameters in SGA and AGA neonates.

	SGA (n=87)	AGA (n=404)	p-value
GA (weeks)	37.1	38.1	<0.001
BW (gram)	1808	2752	<0.001
PCT (%)	0.21	0.25	<0.001
PDW	11.8	11.2	0.016
MPV (fl)	10.0	9.7	0.077
PLT ($\times 10^3/\mu L$)	21.2	26.1	<0.001

MPV, mean platelet volume; PDW, platelet distribution width; MPV, mean platelet volume; PLT, platelet; PCT, plateletcrit.

Discussion

This large cohort showed no differences in platelet parameters at birth between late preterm and term neonates. Previous reports indicated that PLT and PCT were lower in late preterm neonates compared with term neonates (11,12). Furthermore, they suggested that GA was significantly correlated with PLT, however, in our study, GA did not correlate with PLT (11). Instead, there was positive correlation between BW and PLT in preterm and term neonates by univariate analysis. These differences may be due to differences in sample size and time of sampling. Other research included 129 neonates: 58 late preterm and 71 full term. Moreover, they found that

This page contains no comments

PLT measured using cord blood was associated with GA and BW in late preterm and term neonates. In our study, we used cord blood 1 and venous blood at admission to NICU. 2he mean time of sampling in this study was just under 2 hours after birth (data not shown).

This study also demonstrates that various maternal and neonatal factors affect platelet parameters in late preterm and term neonates. Especially, late preterm and term SGA neonates had lower PLT and PCT compared with AGA neonates as previously described (13,14). Lower PLT in SGA indicates the immaturity of thrombopoiesis. (15,16). Roberts suggested that reduced platelet production was characteristic of preterm neonates and they had fewer circulating megakaryocytes (17). Another study reported that plasma thrombopoietin concentrations were low in thrombocytopenic newborns, indicative of inadequate up-regulation of TPO production (18). Platelets are produced in the fetal liver, with production transferred to bone marrow in the third trimester (19). The liver is one of the first organs to be exposed to growth restriction.

On the other hand, Apgar score at 5 minutes was associated with lower PLT in late preterm and term neonates. Some studies previously showed that low Apgar score at 5 minutes was related to thrombocytopenia (20,21). Mario et al showed that SGA, low Apgar score at 5 minutes, cesarean delivery and lower gestational age were risk factors for thrombocytopenia in preterm deliveries between 27 and 35 weeks of gestation (20). To date, perinatal asphyxia has been associated with thrombocytopenia in late preterm and

Number: 1 Author: faizahbhatti Subject: Sticky Note Date: 1/28/20, 11:09:15 AM

This needs to be clearly stated in the methods.

You also need to diffrentiate which values were collected from cord and which were from venous samples. Were these values compared to one another? If not, it would be vital to know if there is a difference in parameters from these two methods of sampling.

Number: 2 Author: faizahbhatti Subject: Highlight Date: 1/28/20, 11:07:34 AM

term neonates (4). In murine models, it is known that hypoxia affects megakaryocyte progenitors and induces thrombocytopenia (22,23). Castle et al reported that severe hypoxia significantly shortened the survival of platelets in a murine model (24). Boutaybi et al found that low Apgar score at 10 min and high serum lactate were risk factors of early-onset thrombocytopenia (25). However, there are few reports about the relationship between perinatal factors and platelet parameters among large cohorts of premature newborns.[1]

The present study suggested that CAM was associated with MPV in term neonates. However, PROM was not associated with MPV as in a previous study (26). Recently, a retrospective study has reported significant correlation between MPV and intrauterine infection in neonates with thrombocytopenia and leukopenia at birth (18,27). Since CAM was indicative of intrauterine infection, our results are consonant with previous studies. Furthermore, PDW in SGA neonates was significantly higher than those of AGA neonates. This result agrees with a previous study (28). Recently, elevated PDW was associated with poor prognosis of various conditions such as carcinoma and cardiovascular disease (1,29,30). Furthermore, Patrick et al reported that high MPV and PDW showed high specificity in neonates with late sepsis (31).

Number: 1 Author: faizahbhatti Subject: Sticky Note Date: 1/28/20, 11:10:06 AM

Determining exposure to epinephrine and resuscitation may help understand this better. See comment above.

Our study has several limitations. First, we used samples of cord blood and venous blood on admission. The timing of samples might affect platelet parameters. Second, we could not investigate the relationship between platelet parameters and sepsis because few of our preterm infants had sepsis at birth and we did not investigate changes of platelet parameters during the postnatal period. Some studies associated sepsis with elevated MPV in neonates during the postnatal period (31,32).

In summary, this study demonstrates that various maternal and neonatal factors affect platelet parameters in late preterm and term neonates. In particular, SGA and low Apgar score at 5 minutes were associated with lower PLT in late preterm and term neonates. CAM was significantly associated with MPV. Thus, this study demonstrates that different maternal and neonatal complications affect platelet parameters in late preterm and term neonates.

Acknowledgments

We would like to thank Kyohei Miyazaki, Kenichi Sato, Hajime Maeda, and Maki Sato for their productive discussions and comments on the manuscript, as well as for their technical help.

This page contains no comments

References

1) Martin JF, Kristensen SD, Mathur A, Grove EL, Choudry FA. The causal role of megakaryocyte-platelet hyperactivity in acute coronary syndromes. Nat Rev Cardiol. 2012;9(11):658-70.

2) Han L, Liu X, Li H, Zou J, Yang Z, Han J, Huang W, Yu L, Zheng Y,Li L. Blood coagulation parameters and platelet indices: changes in normal and preeclamptic pregnancies and predictive values for preeclampsia. PLoS One. 2014;9(2):e114488.

3) Coban E, Adanir H, Bilgin D. The association of mean platelet volume levels with hypertensive retinopathy. Platelets. 2008;19(2):115-8.

4) Roberts I, Murray NA. Neonatal thrombocytopenia: causes and management. Arch Dis Child Neonatal Ed. 2003;88: F359-364.

5) Catal F, Tayman C, Tonbul A, Akca H, Kara S, Tatli MM, Oztekin O, Bilici M. Mean platelet volume (MPV) may simply predict the severity of sepsis in pretem infants. Clin Lab. 2014;60(7):1193-200.

6) ZhaO D, Qiu G, Luo Z, Zhang Y. Platelet parameters and (1,3)-β-D-gulcan as a diagnostic and prognostic marker of invasive fungal disease in preterm infants. PLoS One. 2015;10(4):e0123907.

7) Aydemir C, Aydemir H, Kokturk F,Kulah C, Mungan AG. The cut-off levels of procalcitonin and C-reactive protein and the kinetics of mean platelet volume in preterm neonates with sepsis. BMC Pediatr. 2018;18(1):253.

8) Mitsiakos G, Papathanasiou AE, Kyriakidis I, Karagianni P, Tsepis K, Tzimou I, Lazaridou E, Chatziioannidis I.Intraventricular Hemorrhage andPlatelet indices in Extremely Premature Neonates. J Pediatr Hematol Oncol. 2016;38(7):533-538.

9) Aksoy HT, Güzoğlu N, Eras Z, Canpolat FE, Uraş N. Mean platelet volume/count and mortality in extremely low birth weight infants of preeclamptic mothers. Clin Invest Med. 2018;41(3):E130-135.

10) Kim JY, Yoon J, Lim CS, Choi BM, Yoon SY. Clinical significance of platelet-associated hematological parameters as an

This page contains no comments

early supplementary diagnostic tool for sepsis in thrombocytopenic very-low-birth-weight infants. Platelets. 2015;26(7):620-6.

11) Alicja W, Agnieszka P, Piotr L, Slawomir R, Barbara KW, Milena D, Robert M. Platelet indices in late preterm newborns. J Matern Fetal Neonatal Med. 2017; 30(14):1699-1703.

12) Wasiluk A, Osada J, Dabrowska M, Szczepański M, Jasinska E. Does prematurity affect platelet indices? Adv Med Sci. 2009; 54(2):253-5.

13) Christensen RD, Baer VL, Henry E, Snow GL, Butler A, Sola-Visner MC. Thrombocytopenia in Small-for-Gestational-Age Infants. Pediatrics. 2015; 135(2):e361-70.

14) Wasiluk A, Mantur M, Kemona H, Szczepański M, Jasińska E, Milewski R. Thrombopoiesis in small for gestational age newborns. Platelets. 2009;20(7):520-524.

15) Fustolo-Gunnink SF, Vlug RD, Smits-Wintjens VE, Heckman EJ, Te Pas AB, Fijnvandaraat K, Lopriore E. Early-Onset Thrombocytopenia in Small-For-Gestational-Age Neonates: A retrospective Cohort Study. PLoS One. 2016; 11(5):e0154853.

16) Lee E, Li8m Z, Malhotra A. Thrombocytopenia in well small for gestational age neonates. Blood Coagul Fibrinolysis. 2019. 30(3):104-110.

17) Murray NA, Watts TL, Roberts IA. Endogenous thrombopoietin levels and effect of recombinant human thrombopoietin on megakaryocyte precursors in term and preterm babies. Pediatr Res. 1998; 43:148–151.

18) Watts TL, Murray NA, Roberts IA. Thrombopoietin has a primary role in the regulation of platelet production in preterm babies. Pediatr Res. 1999; 46(1):28-32.

19) Maruyama H, Shinozuka M, Kondoh Y, Akahori Y, Matsuda M, Inoue S, Sumida Y, Morishima T. Thrombocytopenia in Preterm Infants with Intrauterine Growth Restriction. Acta Med. Okayama, 2008:62(5):313-317.

20) Beiner ME, Simchen MJ, Sivan E, Chetrit A, Kuint J, Schiff E. Risk factors for neonatal thrombocytopenia in preterm infants. Am J Perinatol. 2003; 20(1):49-54

21) Nadkarni J, Patne SK, Kispotta R. Hypoxia as a predisposing factor for the development of early onset neonatal thrombocytopenia. J Clin Neonatol. 2012; 1(3):131-134.

22) Cremer M, Weimann A, Schmalisch G, Hammer H, Bührer C, Dame C. Immature platelet values indicate impaired

This page contains no comments

megakaryopoietic activity in neonatal early-onset thrombocytopenia. Thromb Haemost. 2010; 103:1016–1021.
23) Saxonhouse MA, Rimsza LM, Stevens G, Jouei N, Christensen RD, Sola MC. Effects of hypoxia on megakaryocyte progenitors obtained from the umbilical cord blood of term and preterm neonates. Biol Neonate. 2006; 89:104–108.
24) Castle V, Coates G, Mitchell LG, O'Brodovich H, Andrew M. The effect of hypoxia on platelet survival and site of sequestration in the newborn rabbits. Thromb Haemost. 1988; 59(1):45-48.
25) Boutaybi N, Steggerda SJ, Smits-Wintjens VE, van Zwet EW, Walther FJ, Lopriore E. Early-onset thrombocytopenia in near-term and term infants with perinatal asphyxia. Vox Sang. 2014; 106 (4):361-367.
26) Ekin A, Gezer C, Kulhan G, Avcı ME, Taner CE. Can platelet count and mean platelet volume during the first trimester of pregnancy predict preterm premature rupture of membranes? J Obstet Gynaecol Res. 2015; 41(1);23-28.
27) Chen CY, Essien MD, Johnson AJ, Lee GT, Chou FS. Use of mean platelet volume in the assessment of intrauterine infection in newborns with thrombocytopenia and leukopenia at birth. J Matern Fetal Neonatal Med. 2019; 1-7.
28) Wasiluk A, Mantur M, Kenoma H, Szczepański M, Jasińska E, Milewski R. Platelet indices in SGA newborns. Adv Med Sci. 2011. 20(7):520-524.
29) Zuo X, Kong W, Feng L, Zhang H, Meng X, Chen W. Elevated platelet distribution width predicts poor prognosis in hepatocellular carcinoma. Cancer Biomark. 2019;24(3):307-313.
30) Xie X, Zeng X, Cao S, Hu X, Shi Q, Li D, Zhou S, Gu P, Zhang Z. Elevated pretreatment platelet distribution width and platelet count predict poor prognosis in nasopharyngeal carcinoma. Oncotarget. 2017:8(62):106089-106097.
31) Patrick CH, Lazarchick J. The effect of bacteremia on automated platelet measurements in neonates. 1990; 93(3):391-4.
32) Hanaganahalli SB,Sreeram S, Bompada M, Kuppannagari SK, Suresh PK, Philipose CS. Is MPV a Predictive Marker for Neonatal Sepsis? A Pilot Study. J Pediatr Hematol Oncol. 2018; 40(7):548-552.

This page contains no comments

Figure legends

Figure 1. Correlation between platelet count, plateletcrit, GA, and BW.

R means Spearman's correlation coefficient.

This page contains no comments

Click here to download Figure Figure1 PLT.pdf

Figure 1

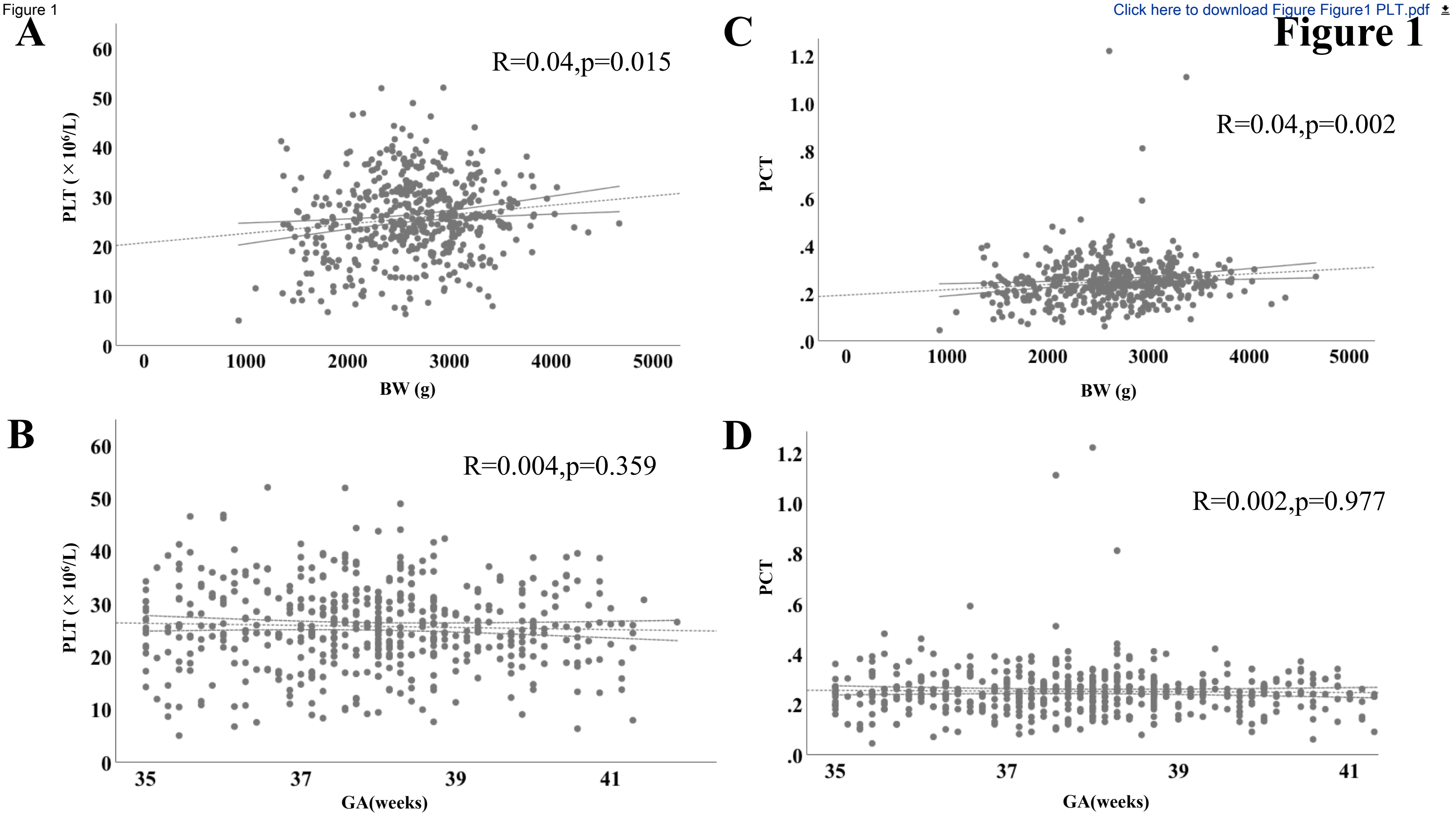

This page contains no comments
