# Supplementary material for: Perinatal factors affecting platelet parameters in late preterm and term neonates
Source: PLoS One. 2020 Nov 19;15(11):e0242539. doi: 10.1371/journal.pone.0242539 (PMC7676724; doi:10.1371/journal.pone.0242539)
Supplement: Attachment — Submitted filename: Summary of Comments on PONE-D-19-33766_reviewer_Bhatti comments.docx [file pone.0242539.s005.docx]

**General Comments:**

This manuscript attempts to provide a cross sectional observation of the platelet’s morphology parameters associated with late preterm and term births. While compelling, the manuscript is lacking in both writing and grammatical issues as well as is lacking in some important analysis. Overall if these issues are addressed this could be an important contribution to the base of knowledge in neonatal intensive care.

The most obvious omission is the lack of connection between the existing base of knowledge on this topic om platelet function and how this would attribute to a clinical outcome. What is the hypothesis? How would a change in platelet number or size influence clinical care?

Better definition of clinical variables needs to be presented. The AGA and SGA descriptors need to be updated. It is important to note in the demographic table 1, what is the proportion of SGA’s in the term and late preterm population.

Specific comments follow below:

**Page: 12**

1. The opening sentence of the Introduction is very vague and not compelling. Please be more specific and deliberate in your writing.
2. What is your hypothesis? It’s not clear what you are adding to the existing body of knowledge.
3. What factors? What comparisons? What are the variables you are interested in studying? At this point, this sounds like an observational study, so please state that explicitly.
4. Why was analysis on preterm babies not performed? That data is discussed in the introduction, but you do not provide a rationale for why it is not included in this study. Why did you limit to term and late preterm?

**Page 13:**

1. When were these samples collected? Beginning of Day of Life 1? At the end? Any time in the first 24 hours? This will confound your results significantly.
2. One of the factors you have failed to examine is how many babies required resuscitation at birth with epinephrine? Epinephrine has been reported to cause platelet aggregation as well as recruitment into the circulation. See Kjeldsen SE, Weder AB, Egan B, Neubig R, Zweifler AJ, Julius S. Effect of circulating epinephrine on platelet function and hematocrit. *Hypertension*. 1995;25(5):1096–1105.
3. Furthermore, reactivity to epinephrine via ADP and thromboxane differs between neonates and adults. Rajasekhar D, Barnard MR, Bednarek FJ, Michelson AD. Platelet hyporeactivity in very low birth weight neonates. *Thromb Haemost*. 1997;77(5):1002–1007.
4. Therefore, it is important to note what the exposure to resuscitation and epinephrine is in these subjects.

**Page 14:**

1. It would have been nice if platelet morphology was examined via microscopy on peripheral smears

**Page 15:**

1. Here it would be very important to separate the term and late preterm subjects by AGA and SGA status. So, have four columns instead of two.

**Page 17:**

1. What is your definition of SGA?

**Page 18:**

1. You have omitted definitions for several of these variables. Is CAM clinical or culture proven? What is the definition of clinical chorio at your institution?

**Page 24:**

1. Timing of sampling needs to be clearly stated in the methods.
2. You also need to differentiate which values were collected from cord and which were from venous samples. Were these values compared to one another? If not, it would be vital to know if there is a difference in parameters from these two methods of sampling.

**Page 25:**

1. Determining exposure to epinephrine and resuscitation may help understand this better. See comment above.
