# Supplementary material for: Perinatal factors affecting platelet parameters in late preterm and term neonates
Source: PLoS One. 2020 Nov 19;15(11):e0242539. doi: 10.1371/journal.pone.0242539 (PMC7676724; doi:10.1371/journal.pone.0242539)
Supplement: Attachment — Submitted filename: Response1030 20201102kn.docx [file pone.0242539.s006.docx]

Nov 3, 2020

Kelli K Ryckman

Editor, *PLoS One*

RE:　Revised Manuscript PONE-D-19-33766

Title: " Perinatal factors affecting platelet parameters in late preterm and term neonates.

"

Dear Kelli K Ryckman

Thank you for your letter of Oct 21, 2020 regarding our manuscript- PONE-D-19-33766. We appreciate the opportunity to respond to the reviewer’s comments.

We wish to express our appreciation to the reviewers and editor for their insightful comments,

which have helped us significantly improve the paper.

We've checked your submission and before we can proceed, we need you to address the following issues:

1) Please justify why the sample size was reduced to 400 from the original 491 in the first submission.

**Response**
Thank you. As we mentioned in the last revision, we found that the original data set included some cases such as congenital heart disease and chromosomal abnormalities. After excluding these cases, the total number of subjects decreased from 491 to 400.

Reviewer #1: Thank you for addressing the comments/reviews you received. Most issues have been adequately addressed.

My only remaining comment is that the results section is still difficult to follow with the abundance of abbreviations being used. Table 2 is also very confusing. Perhaps by reversing the X and Y axis, it will be easier to read and follow. This also applies to Table 3 and 4.

**Response**

Thank you for your suggestion.

We revised Table 2,3,4 so that it will be easier to read and follow.

Reviewer #2:

1.Although, the authors explain that sampling was done in order to exercise caution, it is still unnecessary to do samplings routinely in newborn and the practice is to avoid unless indicated in order to avoid sampling induced blood loss and consequent anemia.

**Response**

Thank you for this comment.

We concur with modern principles of patient blood management. A complete blood count (CBC) is routinely performed on all admissions to NICU, because all of these late preterm and term neonates have various diseases such as jaundice, transient tachypnea of the newborn, pneumothorax, respiratory distress syndrome, hypoglycemia, birth asphyxia, etc. NICU neonates differ from healthy term neonates. Ethically, our abundance of caution includes using blood samples that need to be drawn for immediate NICU care to also collect data that might guide and improve future patient care.

Therefore, we also added the following sentence in discussion.

“Finally, term neonates admitted to our NICU in this study have various diseases such as transient tachypnea of the newborn, pneumothorax, RDS, hypoglycemia, or birth asphyxia. Although RDS is not associated with platelet indices in this study, these term neonates differ from healthy term neonates.”

2. The flow chart has probably typographical error with regards to defining premature birth as > /= 35 weeks .Needs clarification on below which gestation they have defined as premature birth.

**Response**

Thank you for your suggestion. We revised it.

3.Regarding the statistical analysis , the authors need to compile the tables more compressed and crisp so that it is reading friendly. Emphasis should be given to what does it imply . The number of tables should be restricted to 2 or 3 maximum.Rest statistical tables can be added as supplementary material

**Response**

Thank you for your suggestion.

We revised the Tables and convert previous Table 4 and Table 5 to supplemental Table 1 and supplemental Table 2.

4.Odds ratio may be preferred with 95% CI for interpretation of regression analysis

**Response**

Thank you for your suggestion.

Although we could, in principle, add 95% CI in the Table, we have to maintain readability, so in practice, we do not include it.

5.If a complete blood count has also been done along with platelets, then including TLC and ANC as a proxy for sepsis and correlating it with platelet parameters could be of value.

**Response**

While agreeing in principle, we encountered few sepsis cases at birth. Therefore, we could not assess any correlations between platelet parameters and sepsis.

Thank you again for reconsidering our manuscript for publication. We greatly appreciate the reviewers’ comments and suggestions. We are confident that the paper is now greatly improved and we hope that it is now acceptable for publication.

Sincerely,

Hayato Go, MD, PhD

Pediatrics School of Medicine

Fukushima Medical University

1st Hikarigaoka

Fukushima Fukushima Japan, 960-1295

phone#: 81-24-547-1295

FAX#: 81-24-548-6578

E-mail: go@fmu.ac.jp
